# Sorbitol modulates the structure and nanomechanics of κ-casein amyloid fibrils

**DOI:** 10.1016/j.bpj.2026.03.042

**Published:** 2026-03-20

**Authors:** Negar Rahimi, Laura Anasztázia Molnár, Akanksha Sur, Zsombor Dózsa, Petra Molnár, Zsófia Kovács, Sylvio Haas, András Wacha, Dominik Sziklai, Ádám Zolcsák, Barnabás Bőcskei-Antal, Bence Fehér

**Affiliations:** 1 HUN-REN-Semmelweis University Nanobiophysics Research Group, Tűzoltó Street 37-47, 1094 Budapest, Hungary; 2 Institute of Biophysics and Radiation Biology, Semmelweis University, Tűzoltó Street 37-47, 1094 Budapest, Hungary; 3 Deutsches Elektronen-Synchrotron DESY, Notkestrasse 85, 22607 Hamburg, Germany; 4 HUN-REN Research Centre for Natural Sciences, Institute for Materials and Environmental Chemistry, Biological Nanochemistry Group, Magyar tudósok körútja 2, 1117 Budapest, Hungary

## Abstract

Amyloid fibrils are highly ordered protein aggregates that, beyond their pathological roles, are increasingly recognized as functional structures in food systems. κ-casein is a particularly interesting model due to its dual relevance: it stabilizes casein micelles in milk but can also form amyloid fibrils under destabilizing conditions. In this work, we investigate how the food-grade osmolyte sorbitol modulates the fibrillation, structure, and nanomechanics of κ-casein.

Thioflavin T fluorescence assays revealed that sorbitol accelerates fibril formation by eliminating the initial lag phase while maintaining similar elongation kinetics. Atomic force microscopy demonstrated that fibrils formed above 500 mM sorbitol become shorter and significantly stiffer, indicating tighter molecular packing. Complementary small-angle X-ray scattering analysis confirmed that these morphological changes occur without major alterations in the fibril cross-sectional architecture. All-atom molecular dynamics simulations showed that sorbitol induces a redistribution of conformational flexibility, promoting local compaction around central residues and enhanced mobility at the termini, leading to globally more extended conformations.

Together, these results demonstrate that sorbitol acts as a molecular modulator of κ-casein aggregation, linking solution conditions to fibril morphology and nanomechanical properties. These findings provide molecular-level insight into how polyols influence protein self-assembly in food systems and highlight how osmolytes can be used to tune the structural and mechanical properties of amyloid fibrils formed by dietary proteins.

## Significance

Protein aggregation is widely studied in the context of disease, yet amyloid fibrils also play important roles in biological materials and food systems. Understanding how environmental factors regulate fibril formation is therefore essential for controlling protein self-assembly. In this study, we investigate how the food-grade osmolyte sorbitol influences the aggregation of κ-casein, a key milk protein, using fluorescence kinetics, atomic force microscopy, small-angle X-ray scattering, dynamic light scattering, and molecular dynamics simulations. We show that sorbitol modifies early protein assembly in solution and leads to the formation of shorter yet mechanically stiffer fibrils. These findings provide molecular-level insight into how osmolytes tune protein aggregation pathways and the resulting material properties of protein assemblies.

## Introduction

Amyloid fibrils are highly ordered protein aggregates characterized by their cross-β architecture, in which β strands run perpendicular to the fibril axis, and β sheets extend in the direction of fibril growth (1). Although these structures are classically associated with neurodegenerative diseases such as Alzheimer’s and Parkinson’s, amyloid formation also occurs in functional and food-related contexts (2). In food systems, various proteins—including those derived from milk, eggs, and plants—can assemble into amyloid-like fibrillar networks under processing conditions such as heating, acidification, or enzymatic hydrolysis. These fibrillar aggregates influence gelation, texture, water-holding capacity, and stability, positioning amyloid-like assemblies as a promising new class of functional food hydrocolloids.

In addition to their roles in foods, amyloid fibrils have attracted interest as nanoscale building blocks owing to their extreme aspect ratio, mechanical rigidity, and stability (3). Atomic force microscopy (AFM) and other nanomechanical approaches have revealed that amyloid fibrils exhibit elastic moduli ranging from hundreds of MPa to several GPa, comparable to silk or collagen (3,4). This combination of molecular order and mechanical robustness makes amyloid fibrils valuable both as model systems for studying protein self-assembly and as biocompatible materials for structuring foods and designing sustainable biomaterials.

Although amyloid fibrils are often described as intrinsically rigid, their nanomechanical properties are highly sensitive to structural polymorphism and environmental conditions. Single-fibril AFM studies have demonstrated that stiffness can vary by more than an order of magnitude depending on the internal packing within the cross-β core (5,6). Parameters relevant to food processing—such as hydration, temperature, ionic strength, and the presence of small solutes—strongly influence fibril rigidity and assembly kinetics (7). Thus, amyloid fibrils display an adaptable nanomechanical behavior that responds dynamically to their chemical microenvironment, offering molecular-level control over texture and viscoelasticity in protein-based foods.

Solution chemistry plays a central role in regulating amyloid formation, with small solutes known as osmolytes acting as key modulators. Polyols such as sorbitol and glycerol—widely used in the food industry as humectants, stabilizers, and cryoprotectants—affect protein folding and aggregation by altering hydration dynamics and excluded-volume effects (8). Experimental studies have shown that polyols can modify fibrillation kinetics and morphology in proteins such as lysozyme (9). These effects are typically attributed to preferential exclusion of osmolytes from protein surfaces, which modifies water structure and protein-protein interactions (10). Despite this extensive research, the impact of food-relevant osmolytes on the “mechanical” properties of amyloid fibrils remains largely unexplored.

Beyond their technological role in foods, polyols like sorbitol are also physiologically relevant. Sorbitol naturally occurs in human tissues as a metabolic intermediate of glucose and has been shown to influence the organization of biomolecules within cells. Recent studies have demonstrated that sorbitol can enhance liquid-liquid phase separation and promote the formation of biomolecular condensates by acting as a small-molecule crowding agent that modulates protein-protein interactions (11). Such condensate environments lower nucleation barriers and increase local protein concentration—conditions that can facilitate amyloid fibril formation. Therefore, sorbitol-induced changes in the physicochemical surroundings of proteins are likely to affect both the kinetics and the morphology of κ-casein fibrillation.

κ-casein, a glycoprotein responsible for stabilizing casein micelles in bovine milk, represents an excellent model for studying food-related amyloid fibril formation. Under destabilizing conditions such as disulfide bond reduction or heat treatment, κ-casein readily forms amyloid fibrils displaying characteristic cross-β signatures (12,13). Because κ-casein plays a central role in dairy colloid stability and rheology, its conversion into fibrillar aggregates is directly relevant to food texture, digestibility, and storage stability. This duality—being both a natural milk component and a robust amyloid-forming protein—makes κ-casein a unique system for investigating how food-grade additives like sorbitol modulate fibril assembly and mechanical behavior.

Importantly, several digestion-resistant peptides derived from milk proteins, including κ-casein fragments, have been shown to enter the human bloodstream after consumption. It has been demonstrated (14) that κ-caseinoglycopeptide was detectable in human plasma following milk or yogurt ingestion, confirming that certain casein-derived peptides can survive gastrointestinal digestion and cross the intestinal barrier. While the detectability of food-derived peptides in circulation may depend on analytical sensitivity and inter-individual variability (15), their presence indicates that gastrointestinal translocation is possible. Mechanistically, such transport can occur via transcellular or paracellular pathways, particularly under conditions affecting intestinal barrier function (16,17). Milk-protein-derived peptides have also been discussed in relation to barrier-associated and tight-junction pathways in cellular models (18,19).

Although any connection between κ-casein aggregation and clinical phenomena remains speculative, the detection of casein-derived peptides in human circulation suggests that systemic exposure to such fragments is biologically plausible. This raises the possibility that amyloidogenic κ-casein peptides, particularly under conditions of altered intestinal permeability or milk protein intolerance, could interact with biological systems beyond the gastrointestinal tract. Interestingly, κ-casein expression has also been reported in dogs’ mammary tumors (20), where protein misfolding and aggregation processes may influence disease progression. While *in vivo* amyloid formation by κ-casein has not been demonstrated, its well-established amyloidogenic behavior *in vitro* highlights a potential—yet unproven—molecular link between dietary proteins and systemic aggregation pathways.

In this study, we combine thioflavin T (ThT) fluorescence kinetics, AFM, small-angle X-ray scattering (SAXS), and all-atom molecular dynamics (MD) simulations to elucidate how sorbitol—a food-grade osmolyte with physiological relevance—affects κ-casein fibrillation. We show that sorbitol accelerates the onset of fibril formation, reduces fibril length, and significantly increases fibril stiffness, while MD simulations reveal corresponding conformational expansion and rearrangement at the monomeric level. To our knowledge, this is the first report demonstrating that a small-molecule osmolyte can directly modulate the nanomechanical properties of amyloid fibrils. These findings provide molecular-level insight into how osmolytes shape protein aggregation in both food and biological contexts, with implications ranging from dairy protein functionality to potential systemic effects on human health.

## Materials and methods

### Sample preparation

κ-casein fibrils were prepared in phosphate-buffered saline (PBS, 150 mM, pH 7.4). A 0.66 mM κ-casein stock solution was prepared by dissolving 10 mg of protein in 490 μL PBS and stirring at 10°C for 24 h to ensure complete solubilization. To initiate fibrillation, 250 μL of 400 mM dithiothreitol (DTT) and 250 μL of a 4-fold concentrated sorbitol solution were added to the protein stock, yielding a final volume of 1 mL containing 0.33 mM κ-casein, 100 mM DTT, and the desired sorbitol concentration in PBS (0, 250, 500, and 750 mM). The addition of 100 mM DTT reduces intermolecular disulfide bonds in κ-casein, disrupting disulfide-linked oligomers and preventing micelle-like assemblies. Under these reducing conditions, κ-casein predominantly exists in dissociated forms prior to fibrillation, consistent with established protocols for κ-casein amyloid formation (12).

Samples were incubated at 37°C for 24 h without agitation to allow fibril formation. The reaction was stopped by cooling the samples on ice, after which they were stored at 4°C until further analysis.

### Thioflavin T fluorescence assays

Fibril formation was monitored by measuring the fluorescence emission of ThT, a dye that selectively binds to the antiparallel β sheet structures characteristic of amyloid fibrils, including κ-casein fibrils (21).

Emission spectra were recorded at regular time intervals by adding ThT to aliquots withdrawn every 30 min from the incubated κ-casein samples. Measurements were performed at a 100-fold dilution (final κ-casein concentration 0.01%) and a final ThT concentration of 30 μM.

Spectra were collected on an Edinburgh Instruments FLS980 spectrofluorometer using an excitation wavelength of 445 nm and recording emission between 455 and 650 nm (step size 1 nm, integration time 0.4 s). Both excitation and emission slits were set to 2 nm. A xenon arc lamp served as the excitation source, and a red PMT detector was used for emission detection.

Fluorescence intensity maxima were observed at 480 nm for all samples. For each spectrum, the mean emission intensity between 475 and 485 nm was calculated. Corrected, normalized intensity values were obtained by subtracting the initial intensity and dividing by the maximal fluorescence. These normalized intensities were plotted as a function of incubation time to construct ThT kinetic curves.

The reported uncertainties of the fitted parameters correspond to the standard errors obtained from the nonlinear least-squares fitting procedure implemented in OriginPro.

### ANS binding assay

To monitor changes in surface hydrophobicity during κ-casein fibrillation, 8-anilino-1-naphthalenesulfonic acid (ANS) binding assays were performed. ANS was added to the protein samples at a final concentration of 50 μM. Fluorescence measurements were carried out using the earlier described spectrofluorometer setup at 37°C. The excitation wavelength was set to 388 nm, and the emission was first recorded between 410 and 600 nm; after that, we calculated the average intensity out of the range 464–484 nm. Measurements were taken at 15-min intervals for 8 h. The initial time was defined as the addition of the DTT into the samples. To ensure comparability, fluorescence intensities were normalized to the initial value of the reference (0 mM sorbitol sample). Data were smoothed using a 3-point moving average to improve the signal-to-noise ratio.

### Dynamic light scattering

Dynamic light scattering (DLS) measurements were performed to determine the hydrodynamic diameter of κ-casein assemblies prior to fibril formation. Samples were prepared under the same conditions as described above and measured immediately after mixing κ-casein with DTT and sorbitol in order to characterize the initial size distribution before fibrillation. Each measurement represents the average of three runs. All solutions were filtered through 0.22-μm syringe filters prior to measurement to remove dust particles.

Measurements were carried out using a Litesizer 500 instrument (Anton Paar, Austria) at 20°C using a low-volume Uvette measurement cell. Each sample was measured in three independent runs, with an acquisition time of 1 min per run. Data acquisition and analysis were performed using the Kalliope software (Anton Paar). The hydrodynamic diameter (d_H_) was calculated using the cumulant analysis method implemented in the software.

For data analysis, a refractive index of 1.450 and an absorption index of 0.001 were used for the protein, while the solvent (PBS) was defined with a refractive index of 1.3322 and a viscosity of 0.0010157 Pa·s.

### Atomic force microscopy

For AFM imaging, κ-casein fibril samples were diluted 1:1,000 in PBS (150 mM, pH 7.4). A 100-μL droplet was placed on freshly cleaved mica and incubated for 30 min to allow fibril adsorption. The surface was then rinsed gently ten times with 100 μL PBS to remove unbound protein. All steps were carried out at room temperature.

AFM imaging and force spectroscopy were performed on a Nanosurf Drive instrument using BL-AC40TS silicon nitride cantilevers (nominal spring constant 90 pN/nm, tip radius 8 nm). Fibrils were first imaged in WaveMode at a scan size of 1 1 μm to determine fibril length. Image analysis was conducted using Nanosurf Studio and ImageJ; 250 fibrils were measured per condition. Imaging was performed in PBS at 25°C.

For stiffness measurements, force maps (100 × 100 points) were recorded over the same 1 × 1 μm area. Curves were collected with a force setpoint of 2 nN, travel distance of 50 nm, and approach velocity of 1 μm/s. Each curve contained 1,250 points, and indentation depth was maintained below 15% of fibril height. Only curves exhibiting elastic behavior were analyzed.

The Young’s modulus (*E*) was determined by fitting the approach portion of force-indentation curves with the Harding-Sneddon model for a conical indenter:

(1) $$F=\frac{2E}{\pi \left(1-\nu^{2}\right)}\tan\left(\alpha \right)\delta^{2}\text{,}$$

where *ν* = 0.5 is Poisson’s ratio for hydrated proteins, α is the tip half-opening angle (17.5^o^), and *δ* is the indentation depth. Fittings were restricted to forces below 1 nN to satisfy elastic model assumptions. For the fibril length measurements, we only included fibrils longer than 50 nm in the analysis. For the stiffness determination, we matched the force map with the AFM image and only included those force map pixels in the analysis that belonged to fibrils longer than 50 nm and had at least 5-nm indentation depth.

### Statistical analysis of AFM data

Statistical analyses were performed using custom Python 3.12 scripts employing the NumPy, Pandas, SciPy, statsmodels, matplotlib, and seaborn libraries. For each sorbitol concentration (0, 250, 500, and 750 mM), fibril length distributions were examined. After exclusion of missing or nonnumeric values, the right-skewed distributions were log-transformed to approximate normality.

Pairwise comparisons between groups were performed using Welch’s two-sample *t* test (unequal variances). Bonferroni correction was applied to all pairwise *p*-values to account for multiple testing. Differences were considered statistically significant at *p* < 0.05 after correction. Statistically significant differences between groups were indicated with connecting brackets annotated with adjusted *p*-values.

### Small-angle X-ray scattering

SAXS measurements were performed at the SAXSMAT beamline P62 of PETRA ∼ III (DESY, Hamburg, Germany) (22). The X-ray energy was 12.4 keV, and scattering patterns were recorded on an Eiger2∼X∼9M detector at a sample-to-detector distance of 12.6 m, calibrated with collagen. The accessible *q*-range was 0.021–1.10 nm^−1^.

To avoid radiation damage, a flow-through Kapton capillary cell connected to a syringe pump was used. For each sample, 100 frames of 100-ms exposure were recorded and averaged. Between exposures, the sample position was shifted to ensure a fresh, unirradiated volume. The 2D images were azimuthally integrated, normalized to photon flux and transmission, and background-subtracted to yield 1D scattering profiles.

The scattered intensity was plotted as a function of the scattering vector magnitude:

(2) $$q=\frac{4\pi }{\lambda }\sin\;\theta \text{,}$$

where *λ* is the wavelength of the incident radiation, and 2*θ* is the scattering angle.

Experimental data were modeled with Guinier-Porod model described by Hammouda (23). In the high-*q* region, the scattering follows a power law:

(3) $$I_{PL}\left(q\right)=Aq^{-\alpha }\text{,}$$

where *A* is the amplitude, and *α* is the Porod or fractal exponent.

In the low-*q* regime, the generalized Guinier form is used:

(4) $$I_{\text{CG}}\left(q\right)=I_{0}q^{d-3}\;\exp\left(-\frac{q^{2}R_{g}^{2}}{d}\right)\text{,}$$

where *I*_0_ is the forward scattering intensity, *R*_g_ is the radius of gyration, and *d* is the dimensionality parameter: *d* = 3for 3D objects (“normal” radius of gyration), *d* = 2 for cross-sections, and *d* = 1 for thickness. At the crossover point *q*^∗^, the intensity and its derivative are continuous:

(5) $$I_{\text{PL}}\left(q^{*}\right)=I_{\text{CG}}\left(q^{*}\right)$$

(6) $$\frac{\partial I_{\text{PL}}\left(q\right)}{\partial q}\left(q^{*}\right)=\frac{\partial I_{\text{CG}}\left(q\right)}{\partial q}\left(q^{*}\right)$$

Solving these two constraints for the crossover momentum yields

(7) $$q^{*}=\sqrt{\frac{\left(d+\alpha -3\right)d}{2R_{g}^{2}}}$$

Once *q*^∗^is known, Equation 5 may be used to express either *A* from *I*_0_ or vice versa, thereby eliminating two fit parameters per crossover point.

### Molecular dynamics simulations

All-atom and coarse-grained MD simulations were carried out using GROMACS∼2023.2 (24). All-atom systems were constructed with CHARMM-GUI (25), and the initial protein structure was predicted by AlphaFold2 (26). The simulation box extended 3 nm from the protein surface, and the CHARMM36 force field was employed (27). Sodium ions were added to neutralize the system, and further sodium and chloride ions were added to achieve 150 mM salt concentration. Similarly, sorbitol parameters were generated by CHARMM-GUI and inserted into the system to achieve 250, 500, and 750 mM cosolute concentrations.

Energy minimization (5,000 steps, steepest descent) was followed by NVT and NPT equilibration steps (both 125 ps, 1-fs timestep). Long-range electrostatics were computed using the reaction-field method with a 1.2-nm cutoff (28). van der Waals interactions used the same cutoff. Temperature coupling was maintained with a v-rescale thermostat (29) (*τ*_*T*_ = 1 ps) at 280, 300, and 320 K, while pressure was isotropically controlled by the C-rescale barostat (30) (*τ*_*P*_ = 5 ps).

Three independent 300-ns production runs (from three independent protein structures predicted by AphaFold2) were performed for each composition (2-fs timestep), resulting in a total of 900 ns per condition. Visualizations and data processing were performed in Python 3.12 using the NumPy, Pandas, and Matplotlib libraries.

Furthermore, three models were generated by AlfaFold2 of fibril-like dimers. Three independent runs were performed at 0, 250, 500, and 750 mM sorbitol concentrations using the same procedure as described above for the monomers.

### Analysis of molecular dynamics data

#### Radius of gyration analysis

The compactness of the protein was quantified by calculating the radius of gyration (Rg) from the MD trajectories using the GROMACS analysis tool. The radius of gyration describes the mass-weighted root-mean-square distance of the atoms from the center of mass of the protein and provides a measure of the overall structural compactness during the simulation. The calculation was performed over the equilibrated portion of the simulations corresponding to 150–300 ns.

#### Solvent-accessible surface area

The solvent-accessible surface area (SASA) of the protein was calculated using the GROMACS tool, which determines the surface area accessible to a spherical probe representing a solvent molecule. In this method, the solvent-accessible surface is defined by rolling a probe sphere over the van der Waals surface of the protein atoms.

The SASA calculation was performed using the protein as the surface group, while the entire system was used as the reference for accessibility calculations. A probe radius corresponding to the default water probe in GROMACS (0.14 nm) was used.

SASA values were computed for each trajectory frame in the equilibrated simulation window (150–300 ns). The resulting time series was analyzed to monitor fluctuations in the solvent exposure of the protein. Average SASA values were compared between the different sorbitol concentrations to determine whether the presence of the cosolvent altered the degree of protein surface exposure.

#### Hydrogen bond analysis

Hydrogen bonds were identified with the GROMACS’ built-in tool. A hydrogen bond was considered to be present when the distance between the donor and acceptor heavy atoms was ≤0.35 nm and the donor–hydrogen–acceptor angle was ≤150^o^ (equivalently, an H–D–A deviation ≤30^o^).

#### Radial distribution function

The spatial distribution of cosolvent molecules around the protein was quantified using the radial distribution function (RDF), *g*(*r*). RDF calculations were performed using the InterRDF module of the MDAnalysis package.

#### Hydration shell analysis

Local water enrichment near the protein surface was quantified from molecular dynamics trajectories using MDAnalysis (31,32). Four simulation systems were analyzed: a sorbitol-free reference system (NoSorbitol) and three systems containing sorbitol at bulk concentrations of 250 mM, 500 mM, and 750 mM.

Distances were grouped into radial shells extending from 0 to 20 Å with a bin width of 1 Å. For each shell, the number of water molecules was divided by the corresponding spherical shell volume to obtain a local water density profile around the protein. A bulk-like water density reference was estimated as the average density of the outermost five radial shells. The local water enrichment was then defined as the ratio between the shell density and this bulk reference density.

To quantify hydration close to the protein surface, enrichment values were averaged over the distance range of 2–4 Å. This quantity was calculated for every trajectory frame, and the mean and standard error of the mean (SEM) were determined across frames.

To enable direct comparison between systems, the resulting values were normalized to the sorbitol-free reference system; i.e., each system’s mean hydration value was divided by the corresponding value obtained for the NoSorbitol simulation. Error bars represent propagated SEM values after normalization.

#### Residue-residue contact analysis

To quantify intermolecular interactions between the two protein chains, residue-residue contacts were calculated from the molecular dynamics trajectories using the MDAnalysis library. The analysis focused on intermolecular interactions. Contacts were identified based on the minimum distance between atoms belonging to residues from the two chains. A contact was defined when at least one pair of atoms from the two residues was within a distance cutoff of 2.5 Å. To avoid overcounting multiple atom-atom contacts within the same residue pair, residue contacts were counted only once per frame. Two classes of residues were analyzed separately to distinguish between different interaction types: hydrophobic residues (ALA, VAL, LEU, ILE, MET, PHE, TRP, PRO) and charged residues (ASP, GLU, LYS, ARG). For each simulation frame, all possible residue pairs between the two chains belonging to the selected residue class were evaluated. If a residue pair satisfied the distance criterion, it was counted as a contact for that frame. The number of unique residue-residue contacts was then recorded for each analyzed frame. The contact analysis was performed over the equilibrated portion of the trajectories, corresponding to 150–300 ns of simulation time. For each simulation condition (0, 250, 500, and 750 mM sorbitol), the time evolution of the total number of residue-residue contacts per frame was computed. The average number of contacts over the analyzed time window was also calculated to quantify the overall interaction strength between the two protein chains.

#### Contact lifetime analysis

To characterize the stability of intermolecular interactions between the two protein chains, the lifetime of residue-residue contacts was analyzed from the MD trajectories. Contacts were defined using the same geometric criterion as in the contact analysis: two residues were considered to be in contact if at least one pair of atoms belonging to the residues was within 2.5 Å. For each frame of the trajectory, residue pairs satisfying the distance criterion were identified. To avoid overcounting multiple atom-atom contacts between the same residues, each residue pair was counted only once per frame. The temporal persistence of each residue-residue contact was then determined by tracking whether the contact remained present in consecutive frames. The contact lifetime was defined as the continuous time interval during which a given residue pair remained in contact without interruption. If a contact disappeared and later reformed, the subsequent occurrence was treated as a new event. The analysis was performed on the equilibrated portion of the trajectories corresponding to 150–300 ns of simulation time. To reduce temporal correlation while maintaining sufficient sampling, every 10th frame of the trajectory was analyzed. The duration of each contact event was obtained by multiplying the number of consecutive frames in which the contact persisted by the effective time spacing between analyzed frames.

### Bioinformatic tools

To place κ-casein in this broader context, we performed a comparative analysis of the bovine caseins using three complementary bioinformatics tools: CIDER (33), ALBATROSS (34), and the D2P2 disorder database (35).

## Results and discussion

### Structural context of κ-casein among the bovine caseins: Predicted physicochemical characteristics

To interpret the fibrillation behavior of κ-casein, it is necessary to consider its specific sequence features within the casein family. Although all major bovine caseins are intrinsically disordered phosphoproteins, they differ in net charge, charge distribution, hydropathy, and post-translational modifications. These differences influence their conformational ensembles, intermolecular interactions, and roles in micelle organization, thereby contributing to their distinct aggregation propensities.

**Figure 1:**
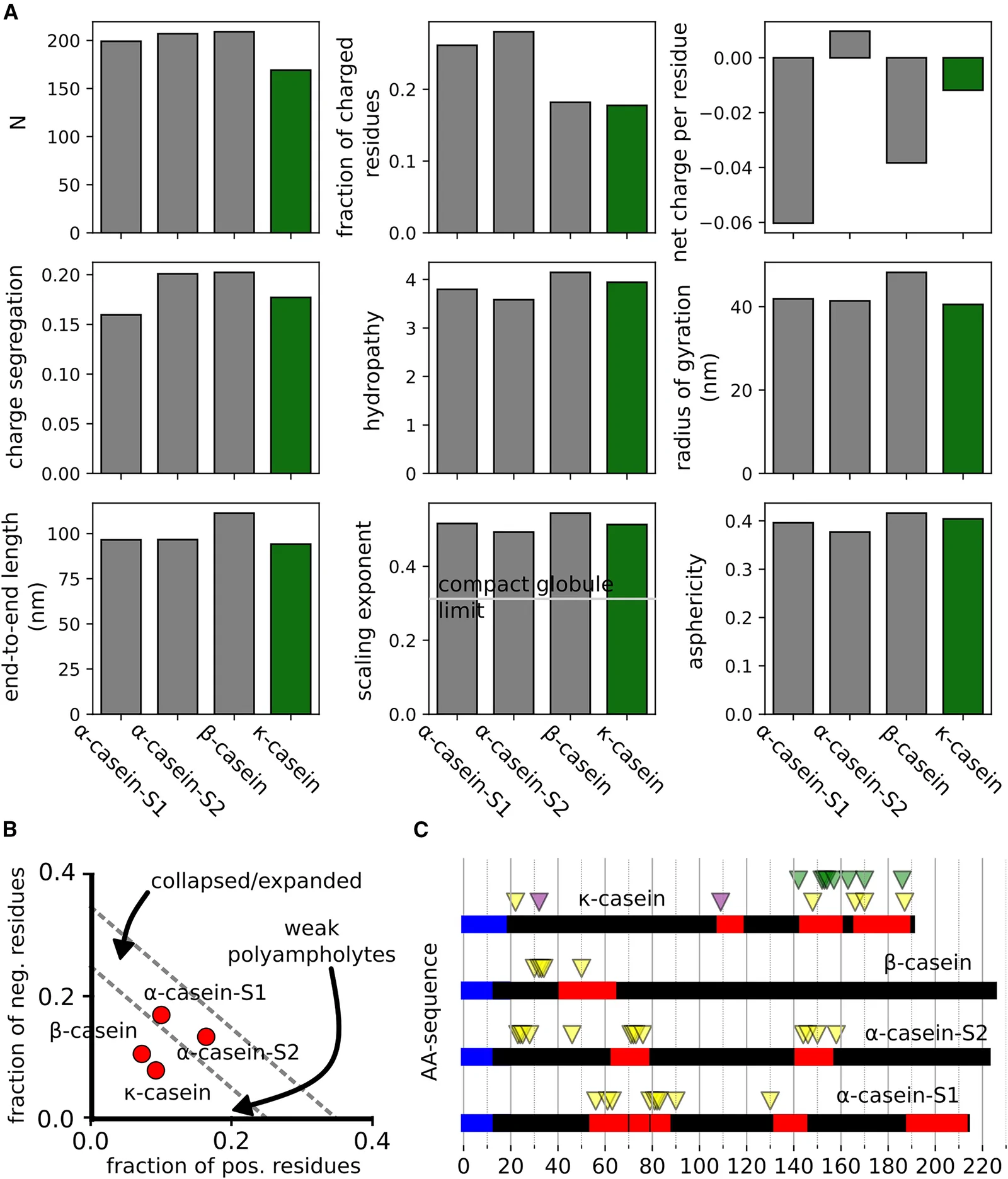
Comparison of caseins based on computational methods. (*A*) Results of the CIDER and ALBATROSS analyses, where N is the number of nonsignal amino acids, and κ is the charge segregation in a sequence. (*B*) Diagram of state classification of the casein proteins. (*C*) Annotated amino acid sequence of caseins, showing how the sequential structure differs in κ-casein. Blue segments: signal region; red segments: consensus D2P2 disorder prediction; yellow arrowheads: modified residues (e.g., phosphorylation); green arrowheads: glycosylation; purple arrowheads: possible disulfide bridges.

Fig. 1 *A* summarizes the results of the CIDER and ALBATROSS analyses. Among the four major bovine caseins, κ-casein is notably smaller (N in Fig. 1) and exhibits a lower fraction of charged residues than *α*_*s*1_- and *α*_*s*2_-caseins, placing it closer to β-casein in terms of net charge and hydropathy. Its net charge is slightly negative but remains close to neutral, suggesting a moderate balance between electrostatic attraction and repulsion. The predicted parameters—radius of gyration, end-to-end distance, scaling exponent, and asphericity—also fall within the range observed for the other caseins.

According to CIDER’s phase diagram of states (Fig. 1 *B*), *α*_*s*1_- and *α*_*s*2_-caseins are predicted to adopt context-dependent collapsed or expanded conformations, whereas β- and κ-caseins behave as weak polyampholytes, tending to form globular or tadpole-like conformations. This global analysis provides an initial structural framework for understanding κ-casein’s behavior but should be interpreted as indicative rather than definitive. A more detailed examination of its sequence features is required to understand its specific physicochemical and functional properties.

The consensus disorder profile obtained from the D2P2 database (Fig. 1 *C*) highlights that intrinsic disorder in κ-casein is predominantly localized in the C-terminal half of the protein. Remarkably, most known post-translational modifications—including phosphorylation and all glycosylation sites—are concentrated within this disordered C-terminal region. In contrast, the N-terminal segment contains two cysteine residues capable of forming inter- or intramolecular disulfide bonds, thereby promoting covalent cross-linking and the formation of intertwined networks or “tadpole-like” assemblies. These features are consistent with the established structural model in which κ-casein forms the stabilizing outer layer of the casein micelle (36).

### Molecular dynamics simulations

#### Monomer simulations

All-atom MD simulations were carried out to investigate the solution behavior of the κ-casein monomer under different sorbitol concentrations. Since it is experimentally challenging to isolate κ-casein monomers, simulations provide valuable insight into the conformational changes that precede fibril formation.

Snapshots from the 300-ns simulations (Fig. 4 *A*) clearly show that the protein adopts a more compact conformation in the absence of sorbitol, whereas the presence of sorbitol results in more extended and flexible structures.

**Figure 2:**
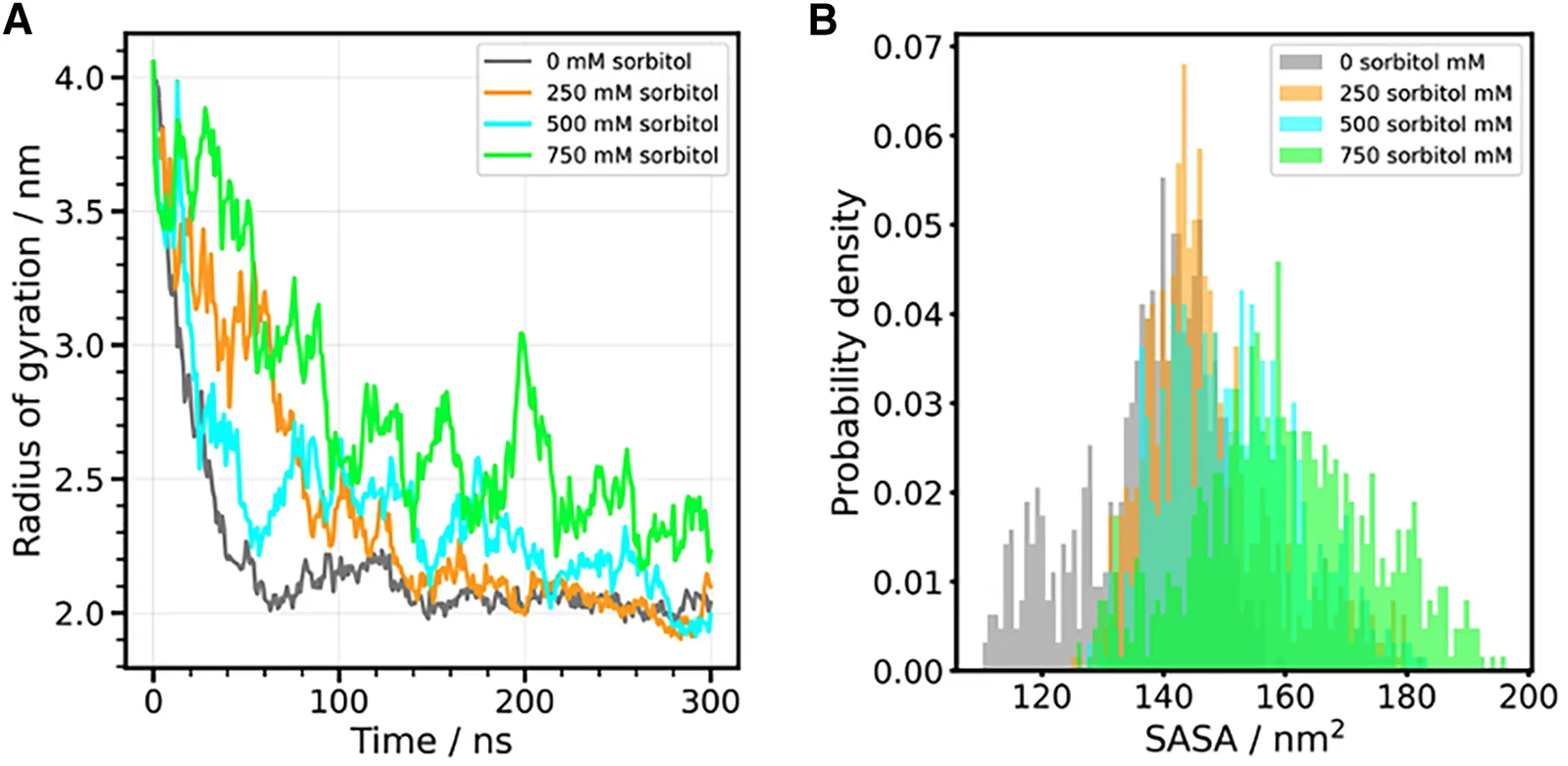
Structural parameters obtained from monomer MD simulations. (*A*) Radius of gyration and (*B*) solvent-accessible surface area of κ-casein at varying sorbitol concentrations (0, 250, 500, and 750 mM).

To quantify these effects, the radius of gyration (*R*_g_) and SASA were calculated from three parallel simulations for each concentration, and their averages are shown in Fig. 2. As seen in Fig. 2 *A*, *R*_g_ converges after approximately 150 ns for all systems, yet fluctuations are more pronounced at higher sorbitol concentrations, especially at 750 mM. SASA histograms (Fig. 2 *B*) reveal a progressive shift toward higher surface areas and broader distributions with increasing sorbitol, indicating that sorbitol promotes more extended conformational states. In contrast, the protein in 0 mM and 250 mM sorbitol remains relatively compact, with a sharp peak around 140 nm^2^. Overall, these observations show that sorbitol shifts the equilibrium ensemble toward more open conformations.

**Figure 3:**
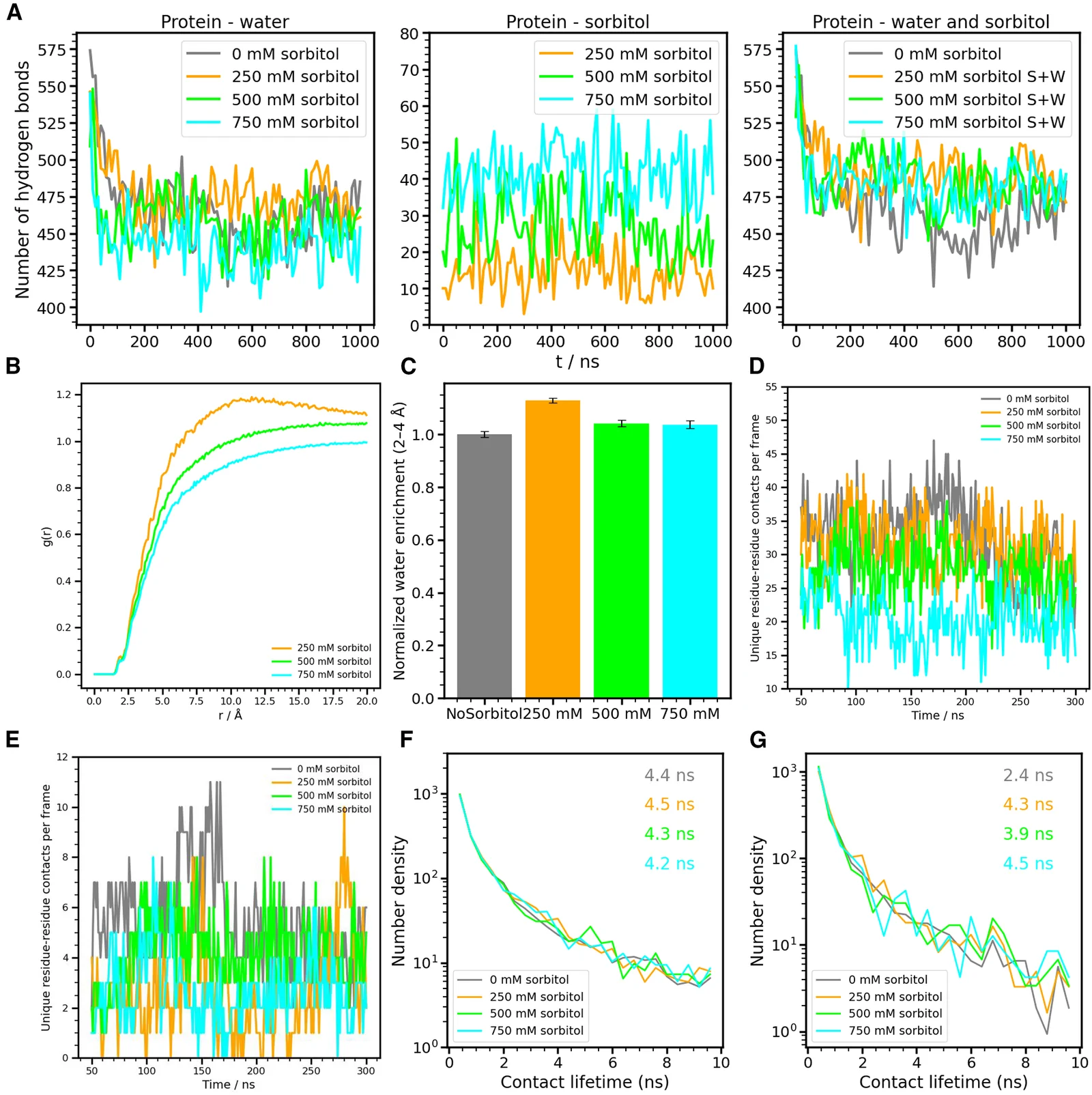
Analysis results of molecular dynamics simulations of monomeric and dimeric κ-casein and water, sorbitol, and their total contributions at different sorbitol concentrations. (*A*) Number of hydrogen bonds between the monomers and solvent; (*B*) radial distribution function between sorbitol and monomer; (*C*) water enrichment around the monomers; (*D*) time evolution of contact numbers between hydrophobic residues of two chains of the dimer; (*E*) time evolution of contact numbers between charged residues of two chains of the dimer; (*F*) contact lifetime between hydrophobic residues of two chains of the dimer; (*G*) contact lifetime between charged residues of two chains of the dimer.

To explore the molecular interactions underlying these conformational changes, we analyzed hydrogen bonding between the protein and the surrounding solvent (Fig. 3 *A*). The number of protein–water and protein–sorbitol hydrogen bonds was determined separately, and their sum was calculated to estimate the total hydrogen-bonding capacity of the system. The number of protein–water hydrogen bonds stabilizes after roughly 60 ns but slightly decreases with increasing sorbitol, reflecting partial replacement of water molecules in the hydration shell. Conversely, protein–sorbitol hydrogen bonds increase proportionally with sorbitol concentration. Interestingly, the total number of hydrogen bonds rises modestly upon the addition of sorbitol but plateaus at higher concentrations. This behavior suggests that sorbitol competes with water for surface sites without continuously increasing the overall hydrogen-bonding capacity, in agreement with preferential exclusion and crowding stabilization mechanisms.

To explore whether sorbitol is indeed excluded from the protein-solvent interface, the radial distribution function between κ-casein and sorbitol was calculated (Fig. 3 *B*). As shown, no sorbitol peak appears close to the protein surface; moreover, the sorbitol concentration is significantly lower near the κ-casein than in the bulk, further supporting the preferential exclusion phenomenon reported in literature data (37,38).

To further quantify how sorbitol affects the local solvent environment of κ-casein, we analyzed the relative enrichment of water molecules in the immediate vicinity of the protein surface. The local water density was evaluated in the 2- to 4-Å hydration shell and normalized to the corresponding value obtained for the sorbitol-free reference simulation (Fig. 3 *C*).

As can be seen, the presence of sorbitol leads to a moderate increase in near-surface water enrichment compared with the reference system. The largest effect is observed at 250 mM sorbitol, where the normalized hydration value exceeds that of the sorbitol-free system by approximately 10%–15%. At higher sorbitol concentrations (500 and 750 mM), the enhancement remains detectable but becomes smaller, indicating a partial saturation of the effect.

These results indicate that sorbitol is preferentially excluded from the immediate protein surface, resulting in a locally water-enriched environment despite the increasing cosolvent concentration in the bulk solution. Such preferential exclusion is consistent with the classical osmolyte mechanism, in which cosolvents destabilize protein-cosolvent contacts relative to protein-water interactions.

The resulting locally water-rich hydration layer may contribute to the expanded conformational ensemble of κ-casein observed in the simulations, as increased hydration favors solvent-exposed protein configurations.

#### Dimer simulations

To gain insight into how the presence of sorbitol influences the first step of the aggregation pathway, dimers were also simulated using all-atom MD. To investigate the intermolecular interactions, the contacts between the two chains were analyzed as described above. To decouple the contribution of hydrophobic and electrostatic interactions, we performed the analysis between hydrophobic and charged residues separately. The time evolutions of the contact numbers of hydrophobic and charged residues are presented in Fig. 3, *D* and *E*, respectively. As shown, sorbitol has no effect on the charged residue-residue contacts. However, a minor decrease between the hydrophobic residues can be observed with increasing sorbitol content. On the other hand, the interpretation of time evolution data might be misleading. In terms of interactions, contact lifetime is more insightful. Therefore, we calculated these values for hydrophobic and charged contacts separately (Fig. 3, *F* and *G*). The contact lifetimes in each case are in the regime of a few nanoseconds, which suggest a very dynamics system with transient contacts. The hydrophobic contact lifetime does not change with increasing sorbitol content. Interestingly, adding sorbitol to the system increases the charged contact lifetime.

To summarize, dimer simulations elucidated that sorbitol invokes more contacts of charged residues, suggesting that sorbitol strengthens the interaction between κ-casein molecules.

**Figure 4:**
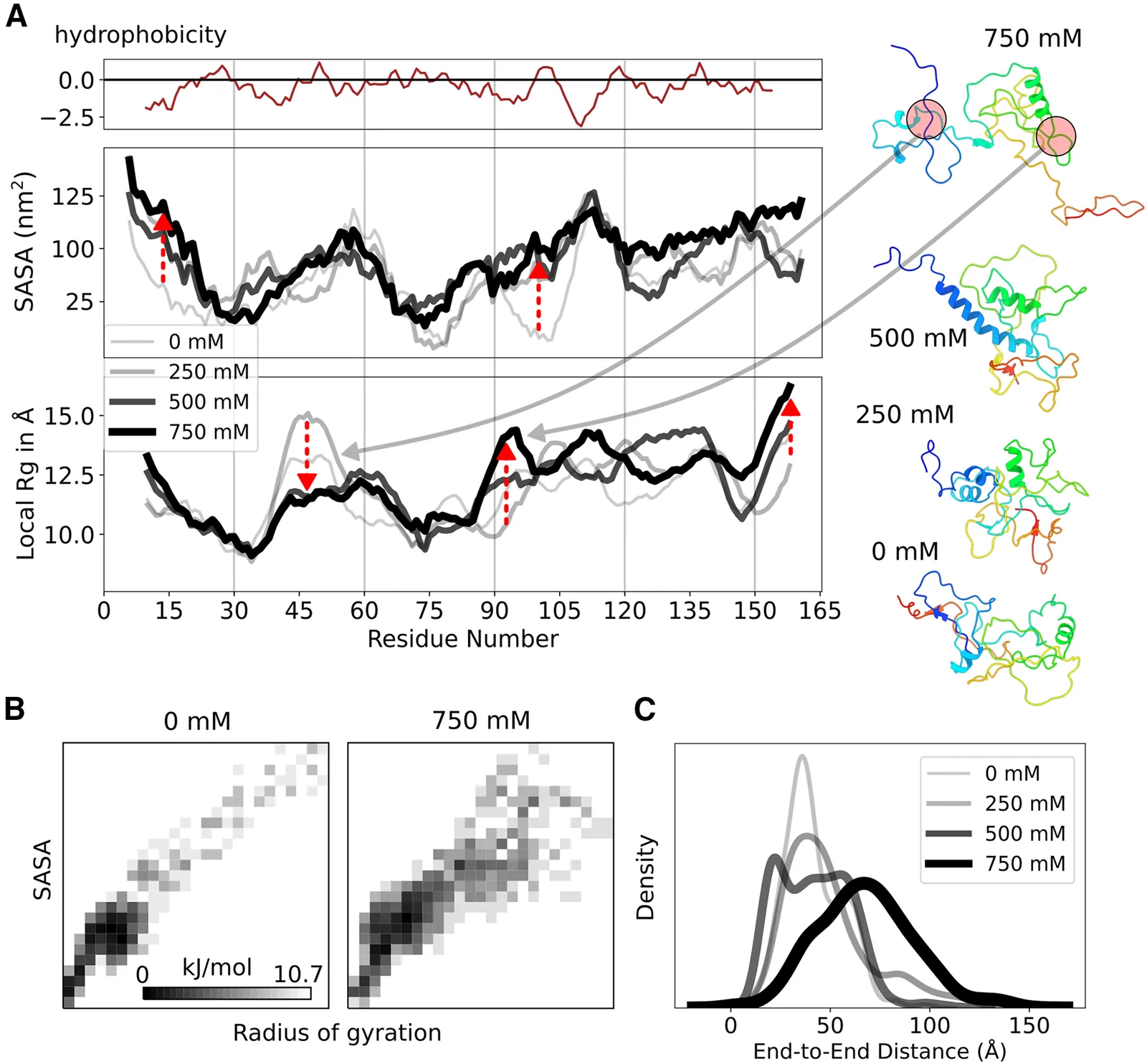
Analysis of all-atom molecular dynamics simulations of κ-casein. (*A*) Hydrophobicity, solvent-accessible surface, and local radius of gyration along the sequence. Red arrows mark key regions showing concentration-dependent shifts. Right: representative final conformations at 0, 250, 500, and 750 mM sorbitol, colored from blue (N terminus) to red (C terminus). (*B*) Gibbs free-energy surfaces for 0 and 750 mM sorbitol as functions of the global radius of gyration and SASA. (*C*) End-to-end distance distributions from the four MD simulations.

The broader conformational landscape of κ-casein at increasing sorbitol concentrations is illustrated in Fig. 4. The free-energy surfaces (Fig. 4 *B*) shift toward higher *R*_g_ and SASA values, confirming the emergence of more open conformations at higher sorbitol concentrations. Consistently, the end-to-end distance distributions (Fig. 4 *C*) show that 750 mM sorbitol favors extended states, whereas 0 mM sorbitol confines the protein to a narrower ensemble of compact configurations.

Residue-level analysis provides additional detail on how sorbitol modulates local flexibility (Fig. 4 *A*). Around residue 45, both local SASA and local radius of gyration decrease with increasing sorbitol, indicating localized compaction. In contrast, residues near 100 and both termini exhibit increased local *R*_g_ and SASA, signifying enhanced mobility and solvent exposure. Thus, sorbitol induces a heterogeneous response: the central core becomes more compact, while the N- and C-terminal regions gain flexibility.

### Kinetics of fibril formation

**Figure 5:**
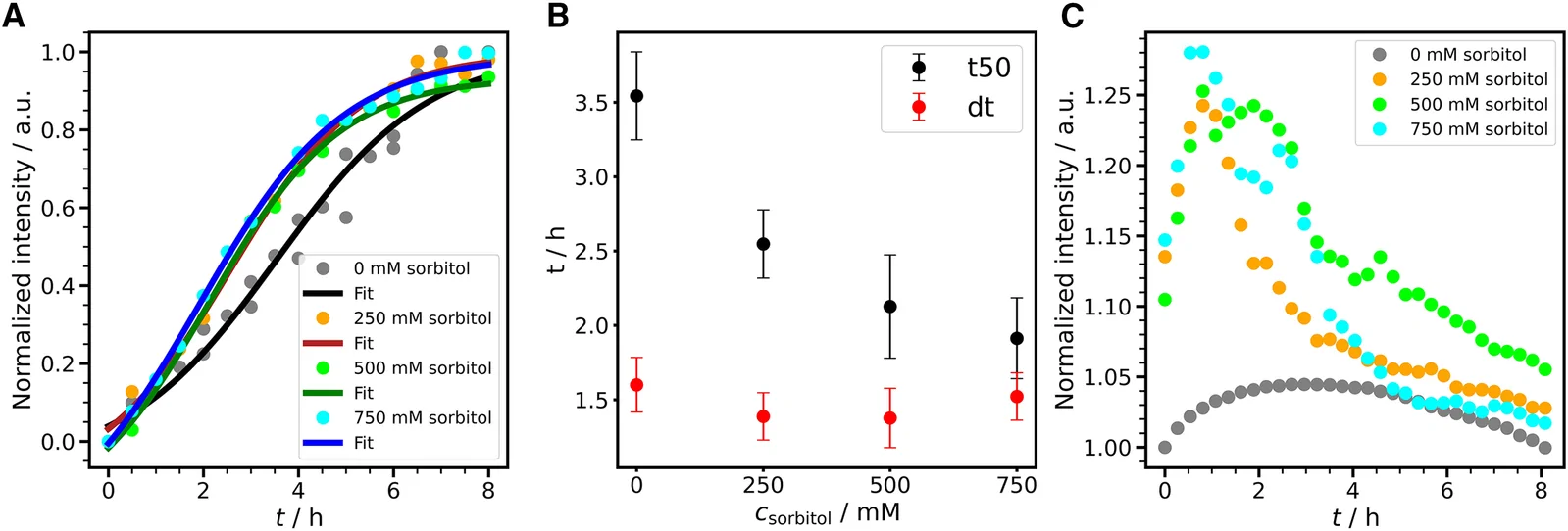
Kinetics of fibril formation followed by the ThT assay. (*A*) Time evolution of fluorescence and fitted curves; (*B*) derived parameters from the fit; (*C*) Chain opening monitored by ANS fluorescence.

To reveal the kinetics of fibril formation, we tracked the evolution of fluorescence using the ThT assay. Fig. 5 *A* shows the normalized scattering intensity as a function of incubation time for various sorbitol concentrations. The time point *t* = 0 corresponds to the initial state of κ-casein fibril formation, whereas the final state represents the point at which all native κ-casein molecules have transformed into fibrillar structures. In practice, the transition between these two states is typically described by a sigmoidal function. In the present study, the kinetic transition was fitted using a modified Boltzmann sigmoid function (continuous lines in the figure):

(8) y=I0+Isat1+et−t50dt+Isat,

where *I*_0_ is the initial intensity, *I*_sat_ is the saturation intensity, *t*50 denotes the time required to reach 50% of the total intensity change between *I*_0_ and *I*_sat_, and d*t* is the slope factor, which reflects the rate of fibril formation. A higher d*t* value corresponds to a slower transition, whereas a lower d*t* indicates a faster process.

The kinetic curves reveal that the fluorescence evolution of the sample without sorbitol differs markedly from that of the sorbitol-containing samples. In contrast, no substantial differences were observed among the samples containing sorbitol at different concentrations. The final fluorescence plateau values were comparable across all sorbitol concentrations within experimental error, indicating that sorbitol does not significantly interfere with ThT binding under the experimental conditions used.

Analysis of the fitted kinetic parameters (Table 1) provides further insight. The slope factor *dt*, which is inversely proportional to the rate of fibril growth, remained constant within experimental error across all sorbitol concentrations. In contrast, the *t*_50_ values show a clear dependence on sorbitol concentration: the sample without sorbitol is distinctly separated from the sorbitol-containing samples.

**Table 1:** Parameters obtained from least-Squares Fitting of Thioflavin T assays

*c*_sorbitol_ (mM)	*t*50 (hours)	d*t* (hours)	Initial state	End state
0	3.54 ± 0.295	1.60 ± 0.183	−0.0664 ± 0.0724	1 ± 0
250	2.54 ± 0.229	1.38 ± 0.159	−0.121 ± 0.0741	0.995 ± 0.0174
500	2.12 ± 0.347	1.37 ± 0.199	−0.219 ± 0.109	0.933 ± 0.0159
750	1.91 ± 0.272	1.52 ± 0.159	−0.288 ± 0.105	0.990 ± 0.0164

These results indicate that κ-casein fibrillation exhibits an initial lag phase in the absence of sorbitol, which is effectively eliminated when sorbitol is present. Since *dt*, which governs the propagation phase, remains nearly identical for all samples, fibril growth proceeds at a comparable rate once nucleation has occurred. This suggests that sorbitol primarily affects the nucleation stage of κ-casein fibrillation, while the elongation rate of existing fibrils remains largely unchanged. Such behavior is consistent with established kinetic models of amyloid fibrillation, in which environmental factors frequently influence nucleation barriers but have a smaller effect on fibril elongation once nuclei have formed (39).

To gain deeper insight into the structural transitions of κ-casein during sorbitol-induced fibrillation, ANS binding assays were employed to monitor changes in surface hydrophobicity (Fig. 5 *C*). The sorbitol-treated samples exhibited a significant initial fluorescence increase—a “hydrophobic burst”—immediately following the reduction of the protein. This suggests that sorbitol facilitates a rapid conformational change, increasing the exposure of hydrophobic regions of the κ-casein monomers. In contrast, the control sample (0 mM sorbitol) showed no such initial jump, displaying a much slower and more gradual increase in surface hydrophobicity over time.

Following the initial burst in the sorbitol-containing samples, a progressive decrease in normalized fluorescence intensity was observed. This downward trend is consistent with the burial of previously exposed hydrophobic patches as they become sequestered within the growing fibrillar core. The rate of this hydrophobic burial closely mirrored the propagation phase observed in the ThT kinetic assays. These results confirm that sorbitol promotes fibrillation by accelerating the initial nucleation through the rapid exposure of hydrophobic domains, which subsequently consolidate into mature, hydrophobic-core-containing fibrils.

**Figure 6:**
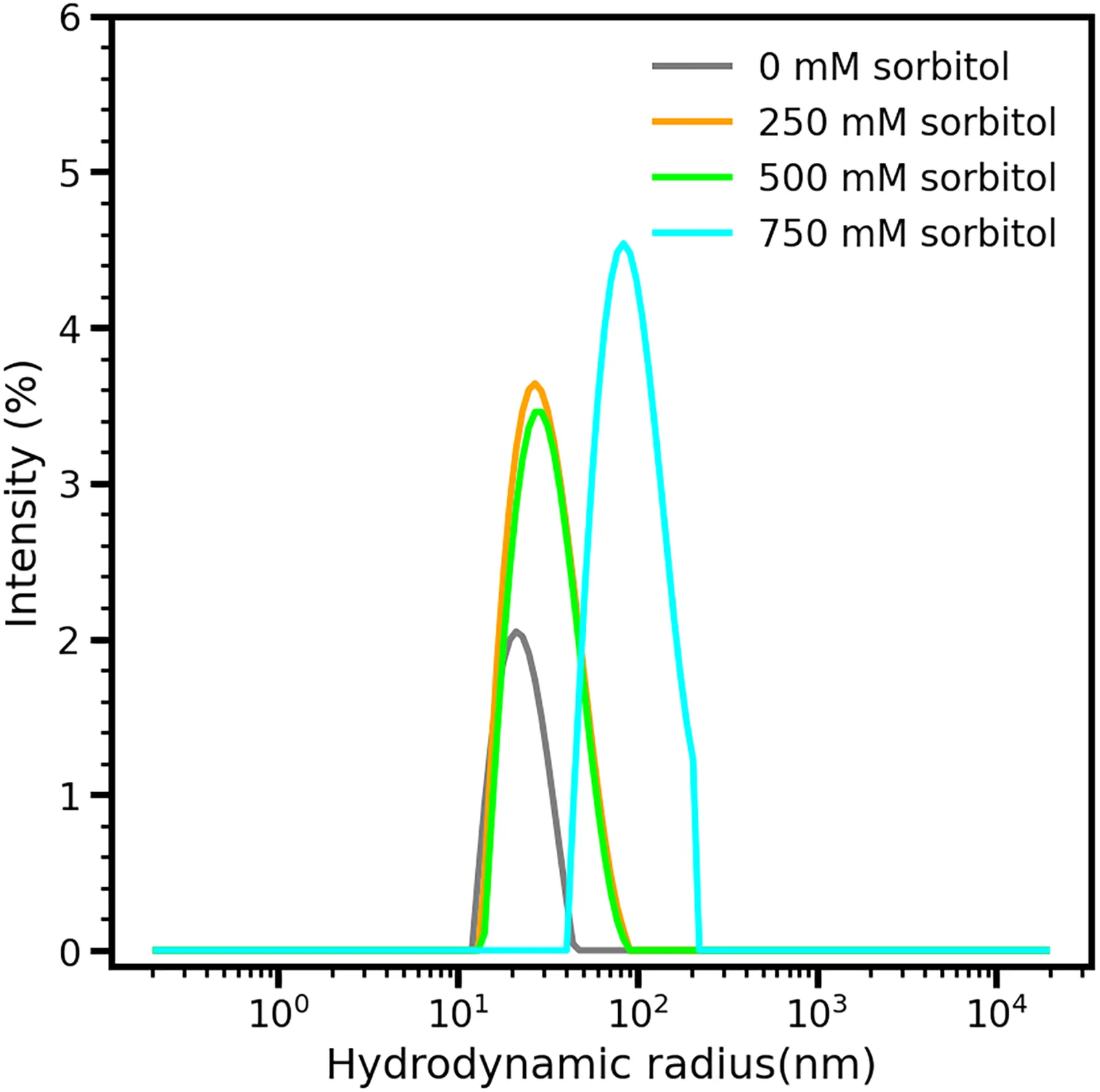
Intensity-weighted size distribution of κ-casein at different sorbitol concentrations at 0 time point, obtained by dynamic light scattering.

As the ThT assays suggest the absence of a lag phase in sorbitol-containing samples, we performed DLS on the initial solutions at 0 time point (before incubation) to reveal the nucleation state at the beginning of the fibril-forming process (Fig. 6). It can be clearly seen that, with increasing the sorbitol content, the position of the peak shifts toward larger hydrodynamic diameters. We are aware that DLS can be only used for well-defined spherical objects; moreover, in 750 mM sorbitol, a shoulder appears in the distribution. At the same time, at the nucleation stage, small aggregates can be treated as sphere-like objects; therefore, the data provide valuable insight, indicating that in the presence of sorbitol, small aggregates are already present. This suggests that the fibrillation process starts immediately upon incubation, effectively “skipping” the lag phase.

### Structure and mechanics of fibrils

**Figure 7:**
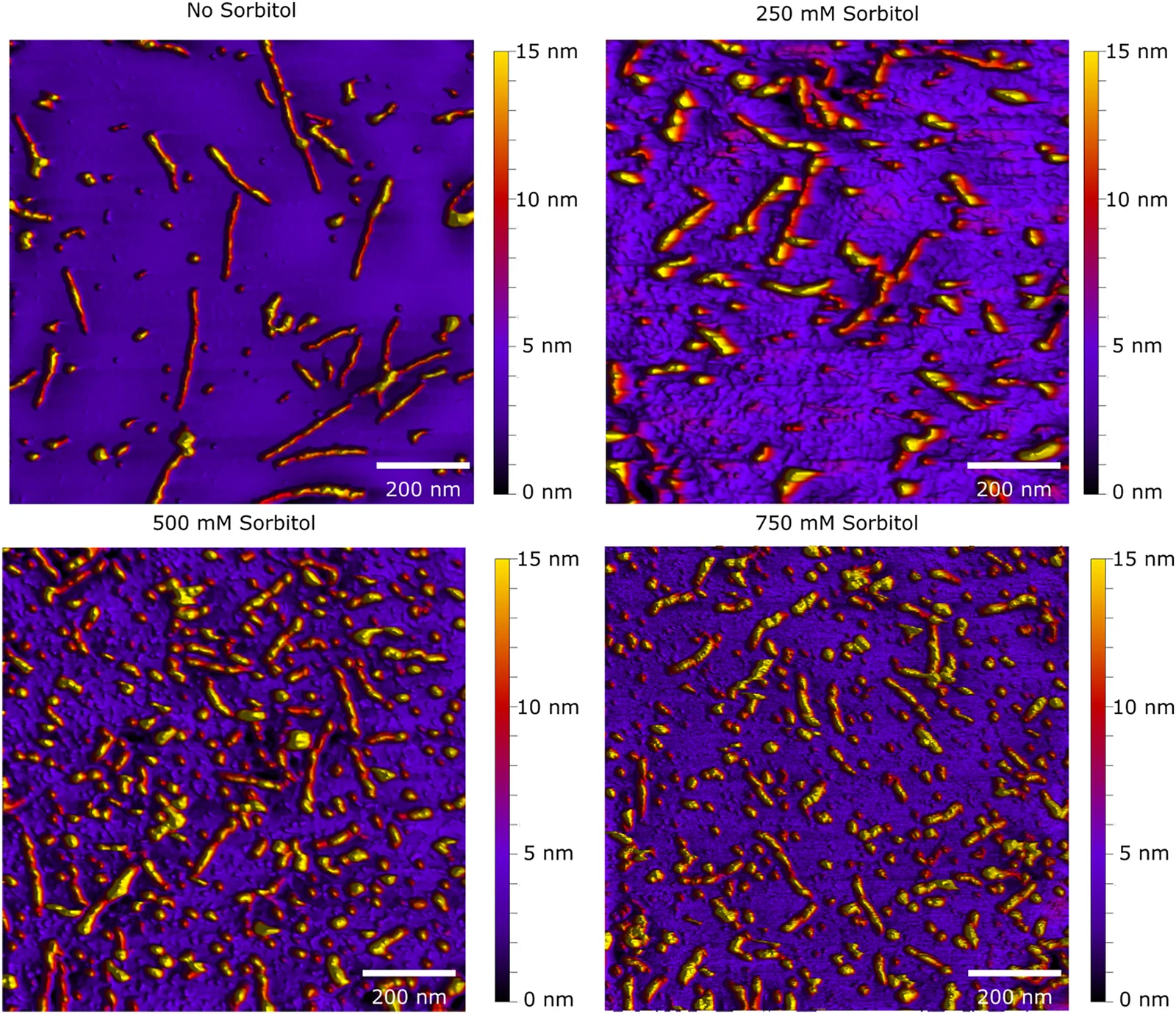
AFM height images of κ-casein fibrils formed at different sorbitol concentrations.

To investigate the morphology and nanomechanical properties of κ-casein fibrils, AFM measurements were performed under varying sorbitol concentrations (0, 250, 500, and 750 mM). AFM imaging revealed that fibrils formed under all tested conditions (Fig. 7). Visual inspection of the AFM topographs showed that increasing sorbitol concentration induced a transition toward shorter and more uniform fibrillar morphologies, while also favoring the appearance of irregular aggregate species. To quantitatively confirm this observation, the lengths of 250 individual fibrils were measured per sample, and statistical analysis was performed using Bonferroni-corrected Welch’s *t* tests.

As summarized in Table 2, the average fibril length remained nearly unchanged up to 250 mM sorbitol. However, further increasing the sorbitol concentration to 500 and 750 mM led to a reduction in fibril length by approximately 20 nm. The statistical results indicate a threshold behavior between 250 and 500 mM sorbitol, above which the fibril length decreases significantly, whereas below this threshold, the effect of sorbitol remains negligible.

**Table 2:** Length and Young’s modulus of κ-Casein Fibrils prepared in 0, 250, 500, and 750 mM Sorbitol obtained from the AFM Measurements

	0 mM Sorbitol	250 mM Sorbitol	500 mM Sorbitol	750 mM Sorbitol
Length (nm)	150 ± 57.2	153 ± 55.7	136 ± 40.9	131 ± 43.3
Young’s modulus (MPa)	55.1 ± 16.5	49.7 ± 16.2	66.9 ± 20.5	65.1 ± 23.9

Force spectroscopy measurements further revealed that the stiffness of κ-casein fibrils increased with rising sorbitol concentration. The obtained stiffness values (∼10^7^ Pa) are consistent with earlier AFM studies on amyloid fibrils, which reported elastic moduli in the range of 0.1–10 GPa depending on fibril type and experimental conditions (3,4). Median Young’s modulus values increased by approximately 1.3–1.5 · 10^7^ Pa at 500 and 750 mM sorbitol compared with the control and 250 mM conditions. These increases were statistically significant (*p*-values of 9.87 · 10^−7^ and 4.17 · 10^−5^), whereas no further change was observed between 500 and 750 mM.

Taken together, the AFM results demonstrate that sorbitol induces a concentration-dependent transition in the morphology and mechanics of κ-casein fibrils. Above a critical concentration (approximately 500 mM), the fibrils become simultaneously shorter and stiffer, while further increases in sorbitol do not produce additional changes in either length or stiffness. This suggests that sorbitol modulates the equilibrium between nucleation and elongation processes and promotes denser β sheet packing within the fibrils. Similar osmolyte-induced effects on amyloid fibril growth have been reported for other proteins such as lysozyme and insulin (9).

**Figure 8:**
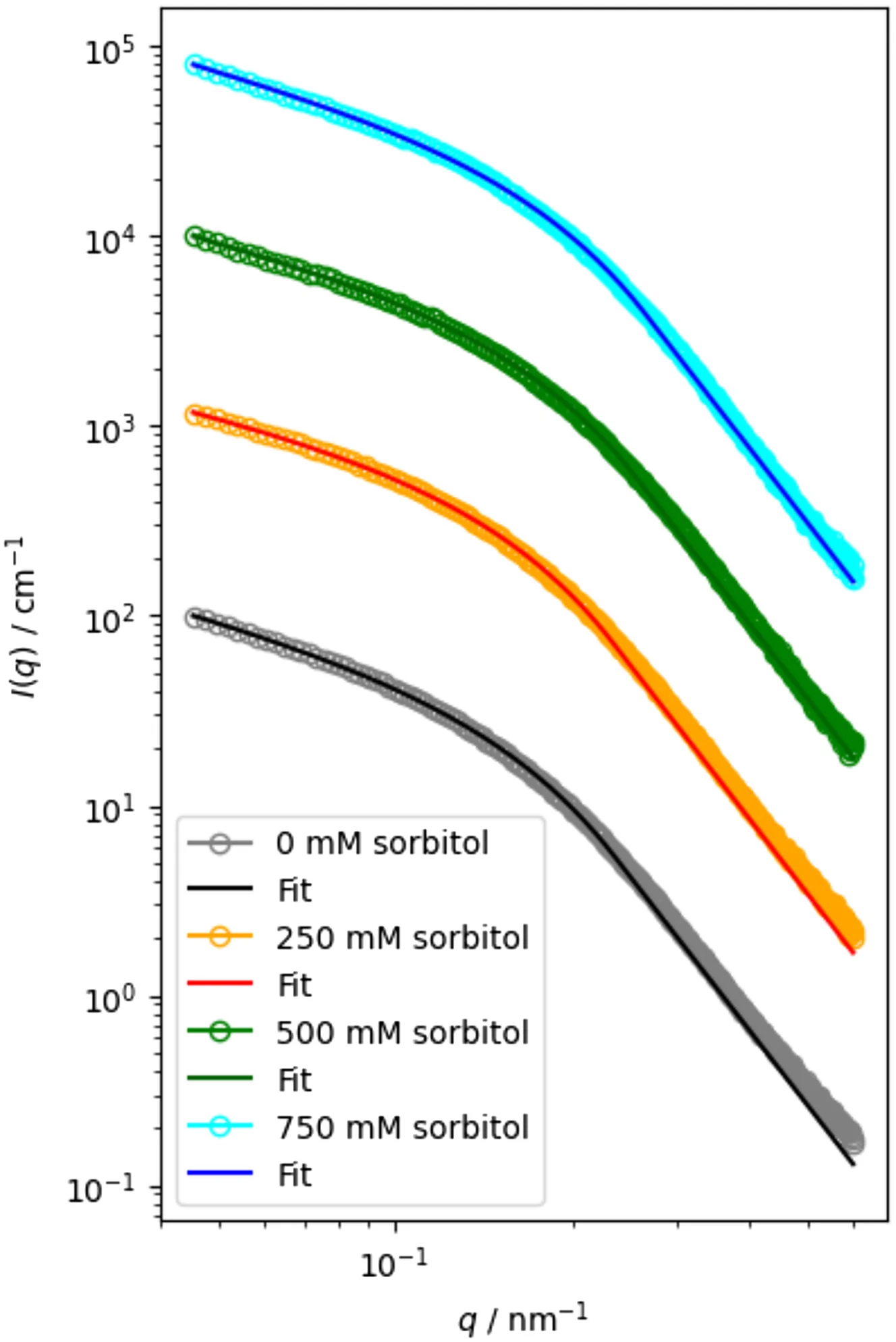
SAXS curves of κ-casein without sorbitol and at 250, 500, and 750 mM sorbitol concentrations. The curves were scaled for better visibility with factors of 1, 10, 100, and 1,000, respectively.

To complement the morphological information obtained by AFM, SAXS experiments were performed on κ-casein fibril samples prepared at different sorbitol concentrations. The resulting scattering profiles are presented in Fig. 8 (intensities are scaled for clarity). The overall scattering features were similar across all sorbitol concentrations. No Guinier plateau was observed in the low-*q* region, and the gradual decrease in intensity suggests that the fibril length exceeds the upper limit of the instrumental resolution range. To gain deeper insight into the nanoscale structure, the SAXS data were analyzed the using Guinier-Porod method. The corresponding fitted curves are also displayed in Fig. 8, and the extracted parameters are summarized in Table 3. According to the fits, the cross-sectional radius of gyration was determined (*d* ∼2), which shows a decreasing trend with increasing sorbitol concentration. The increased compactness of the fibrils is in line with AFM data showing increased stiffness.

**Table 3:** Parameters obtained from SAXS fitting

	0 mM Sorbitol	250 mM Sorbitol	500 mM Sorbitol	750 mM Sorbitol
*logI*_0_	0.884 ± 0.00436	1.11 ± 0.00510	0.966 ± 0.00453	0.787 ± 0.00500
*R*_g_(nm)	7.81 ± 0.0289	8.23 ± 0.0334	7.37 ± 0.0294	6.92 ± 0.0325
*d*	2.15 ± 0.00340	2.26 ± 0.00398	2.21 ± 0.00357	2.15 ± 0.00395

The AFM and SAXS data together provide a consistent picture of κ-casein fibril formation in the presence of sorbitol. AFM revealed pronounced changes in fibril length and stiffness above 500 mM sorbitol, and SAXS confirmed that the fibrils become thinner in the presence of sorbitol. The combination of both techniques therefore suggests that sorbitol primarily affects the supramolecular organization and packing of fibrils—promoting shorter, more compact, and stiffer assemblies.

## Conclusion

In this work, we combined fluorescence kinetics, AFM, SAXS, and MD simulations to elucidate how sorbitol modulates the fibrillation and conformational behavior of κ-casein.

The MD simulations provided a complementary microscopic picture of these processes. Increasing sorbitol concentration induced a redistribution of conformational flexibility. Globally, the conformational ensemble shifted toward more expanded and solvent-exposed states, driven by preferential water-protein interactions and preferential exclusion of sorbitol from the hydration shell; however, the central part of the protein became more compact. These structural rearrangements promote enhanced intermolecular interactions and the formation of compact, mechanically reinforced fibrils observed experimentally.

ThT assays revealed that sorbitol significantly accelerates the onset of fibril formation by eliminating the initial lag phase, while the propagation rate of fibril growth remains largely unaffected.

AFM demonstrated a concentration-dependent transition in fibril morphology and mechanics: above ∼500 mM sorbitol, the fibrils become shorter and stiffer, indicating denser molecular packing. SAXS analysis confirmed that these morphological changes occur without major alterations in the internal cross-sectional architecture of the fibrils.

Together, the experimental and computational results establish a coherent model in which sorbitol acts as a molecular modulator of κ-casein self-assembly.

Beyond its relevance to dairy protein functionality and food formulation, this work provides molecular-level insight into how polyols can modulate aggregation-prone protein ensembles and the resulting fibril nanomechanics. Although the biomedical implications remain speculative, κ-casein-derived peptides have been detected in human plasma after dairy intake (40), and recent studies discuss the variable detectability of food-derived peptides in circulation across individuals and analytical platforms (15). Considering established concepts of peptide stability and intestinal transport mechanisms (16,17), as well as literature linking milk protein derivatives to barrier-associated pathways (18,19), our findings motivate future work to test whether osmolyte-dependent structural changes in dietary protein assemblies have measurable biological consequences under physiologically relevant conditions.

## Data and code availability

Data are available at https://doi.org/10.5281/zenodo.17865741.

## Acknowledgments

This research project was supported by HUN-REN Welcome Home and Foreign Researcher Recruitment Programme 2023. This project was supported by the 10.13039/501100018818National Research, Development and Innovation Office of Hungary under STARTING grant (150679) and under grant FK146081. We acknowledge [KIFÜ] for awarding us access to resources based in Hungary at Komondor for CPU and GPU time. A.W. was supported by the János Bolyai Research Scholarship of the Hungarian Academy of Sciences.

## Author contributions

N.R.: conceptualization, methodology, formal analysis, visualization, and writing – original draft, review & editing. L.A.M.: methodology, visualization, writing – original draft. A.S.: methodology, visualization, and writing – original draft. Z.D.: methodology and visualization. Z.K.: methodology and visualization. S.H.: methodology, visualization, writing – original draft. A.W.: methodology, visualization, and writing – original draft. D.S.: methodology, visualization, and writing – original draft. Á.Z.: methodology, visualization, and writing – original draft. B.B.-A.: methodology, visualization, and writing – original draft. B.F.: principal investigator, conceptualization, supervision, and writing – review & editing.

## Declaration of interests

The authors declare no competing interests.
